# Discrimination exposure impacts unhealthy processing of food cues: crosstalk between the brain and gut

**DOI:** 10.1038/s44220-023-00134-9

**Published:** 2023-10-02

**Authors:** Xiaobei Zhang, Hao Wang, Lisa A. Kilpatrick, Tien S. Dong, Gilbert C. Gee, Jennifer S. Labus, Vadim Osadchiy, Hiram Beltran-Sanchez, May C. Wang, Allison Vaughan, Arpana Gupta

**Affiliations:** 1 G. Oppenheimer Center for Neurobiology of Stress and Resilience, UCLA, Los Angeles, CA, USA.; 2 Vatche and Tamar Manoukian Division of Digestive Diseases, UCLA, Los Angeles, CA, USA.; 3 David Geffen School of Medicine, UCLA, Los Angeles, CA, USA.; 4 University of California, Los Angeles (UCLA), Los Angeles, CA, USA.; 5 Goodman–Luskin Microbiome Center, UCLA, Los Angeles, CA, USA.; 6 Department of Community Health Sciences Fielding School of Public Health, UCLA, Los Angeles, CA, USA.; 7 California Center for Population Research, UCLA, Los Angeles, CA, USA.; 8 Department of Urology, UCLA, Los Angeles, CA, USA.; 9 School of Physics and Optoelectronic Engineering, Hainan University, Haikou, China.

## Abstract

Experiences of discrimination are associated with adverse health outcomes, including obesity. However, the mechanisms by which discrimination leads to obesity remain unclear. Utilizing multi-omics analyses of neuroimaging and fecal metabolites, we investigated the impact of discrimination exposure on brain reactivity to food images and associated dysregulations in the brain–gut–microbiome system. We show that discrimination is associated with increased food-cue reactivity in frontal-striatal regions involved in reward, motivation and executive control; altered glutamate-pathway metabolites involved in oxidative stress and inflammation as well as preference for unhealthy foods. Associations between discrimination-related brain and gut signatures were skewed towards unhealthy sweet foods after adjusting for age, diet, body mass index, race and socioeconomic status. Discrimination, as a stressor, may contribute to enhanced food-cue reactivity and brain–gut–microbiome disruptions that can promote unhealthy eating behaviors, leading to increased risk for obesity. Treatments that normalize these alterations may benefit individuals who experience discrimination-related stress.

Racial disparities in obesity persist in America, with minority subgroups experiencing disproportionally higher rates of obesity and obesity-related morbidities^1–5^. Multiple factors could contribute to such disparities, with the existing literature primarily focused on the role of genetics, diet, physical activity and psychological factors^6^. Despite its relevance to the etiology of obesity, few studies have directly examined the role of discrimination experiences in the pathways that may increase obesity risk.

Discrimination, a type of psychosocial stressor^7,8^, is an environmental risk factor for various adverse health outcomes^9–15^. Experiences of discrimination can stimulate ingestive behavior by increasing appetite, cravings and motivation to consume highly palatable foods, contributing to stress-related weight gain in obesity^16^. Neuroimaging studies indicate that stress can alter food-cue reactivity to highly palatable foods^17,18^. Accordingly, experiences of discrimination may lead to an increased obesity risk via altered food-cue reactivity to hypercaloric and hyperpalatable foods, which are ubiquitous in the Western diet^19^.

One potential mechanism linking discrimination and obesity involves alterations in the brain–gut–microbiome (BGM) system. Individuals who experience discrimination present heightened stress responses^20–22^. Stress in turn influences the bidirectional communication between the brain and gut via pathways involving the vagus nerve, immune-inflammatory mechanisms, altered microbial metabolites, neurotransmitters and the hypothalamic–pituitary–adrenal axis^23–27^.

Brain pathways associated with discrimination-related stress responses include reward and cognitive control networks^7,8,28–33^. A recent study indicated that exposure to discrimination was associated with brain functional connectivity alterations in the central executive network^33^. Overlap in neurobiological pathways linked to stress and energy homeostasis may underlie the co-occurrence of dysregulated feeding behaviors and stress responses, both of which can contribute to obesity^16,34^. Chronic stress alters responses in prefrontal regions associated with executive control and emotion/impulse regulation as well as in limbic regions involved in reward processing and appetitive responses, providing a neural basis for the impact of chronic stress in modulating food-reward processing and cravings^19,35–37^. In response to food cues, stress could result in the deactivation of frontal executive modulation and potentiate brain activity in limbic regions, eliciting a bias towards unhealthy energy-dense foods^17,18^.

Discrimination can also alter the gut microbiome^33^. Stress facilitates gut dysbiosis and increases gut-barrier permeability, producing an inflammatory response and a leaky gut^38^. Stress-induced unhealthy dietary patterns could result in gut dysbiosis and prompt dysregulated eating behaviors catering to the needs of dominant bacterial species^39^. Dysregulation of glutamate metabolism plays an important role in inflammatory processes of the central nervous system (CNS) that are associated with stress-related disorders (for example, depression and anxiety) and obesity^40–43^. A recent study revealed that stress in early life was associated with altered gut metabolites within the glutamate pathway, potentially via glutamatergic excitotoxicity and oxidative stress mechanisms^44–46^. These stress-related gut metabolites were also associated with alterations in the brain functional connectivity involved in cognitive and emotional processes. Glutamate is also involved in executive control and reward processing, two functions that are highly relevant to the processing of food cues^47,48^. Together, these studies highlight the role of altered glutamate metabolites in the stress response and their relevance in brain and gut communication.

In this study we investigated the impact of discrimination exposure on neural reactivity to unhealthy and healthy food cues, relevant gut metabolites as well as brain–gut associations to elucidate potential mechanisms linking discrimination and obesity. We hypothesized that increased stress due to higher levels of discrimination exposure, would be associated with: (1) altered brain reactivity towards highly palatable unhealthy foods in brain regions that are associated with reward processing and executive control and (2) altered levels of glutamate metabolites that are implicated in inflammation and oxidative stress. In addition, given that discrimination may modulate the crosstalk between the brain and gut microbiome system^49^, we predicted that important interactions between discrimination-related neural reactivity to unhealthy sweet-tasting (sugar-rich) foods due to their unique rewarding and analgesic nature^50–53^ and metabolites from the glutamate pathway would be observed (Fig. 1).

## Results

### Participant characteristics

There were no significant differences in sex, age, body mass index (BMI), education, marital status, income or diet between the two discrimination exposure groups but socioeconomic status (SES) was significantly lower in individuals with high discrimination exposure (Table 1). Discrimination and diet did not have interaction effects on BMI.

### Discrimination-related whole-brain analysis

**Fig. 2 |:**
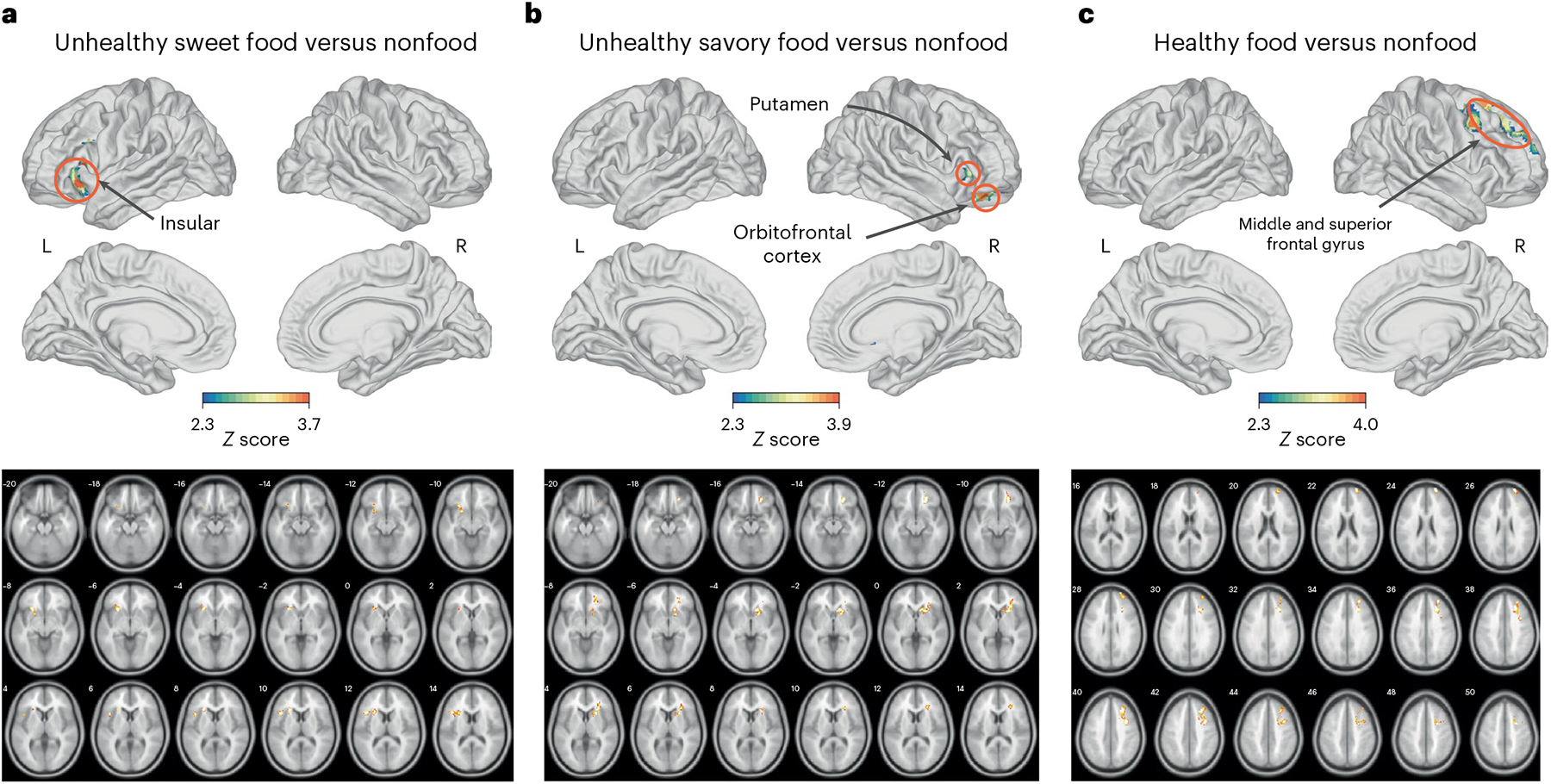
Whole-brain comparisons of the high and low discrimination groups. **a**–**c**, Schematics (top) and MRI images (bottom) of the brains of individuals to compare the food-cue reactivities of unhealthy sweet (**a**), unhealthy savory (**b**) and healthy (**c**) food with nonfood. Regions where greater reactivity was observed in the high discrimination group (*n* = 50) relative to the low discrimination group (*n* = 57) are highlighted (colored region); the color bar represents the *Z* score, with warmer colors indicating higher scores. Comparisons controlled for BMI, sex, age, race, diet and SES. All statistical maps were family-wise error cluster-corrected for multiple comparisons. Cluster level correction: *Z* > 2.3, *P* < 0.05. Clusters are listed in Table 2. The numbers located at the top left of each MRI image represent the slices. L, left hemisphere; R, right hemisphere.

When using magnetic resonance imaging (MRI) to compare responses to unhealthy sweet foods and nonfoods, the group of individuals exposed to discrimination (high discrimination group) had greater food-cue reactivity towards unhealthy sweet foods than the low discrimination group in the insula, inferior frontal gyrus, lateral orbitofrontal cortex and frontal operculum cortex (Fig. 2a).

In the comparison of unhealthy savory foods versus nonfoods, the high discrimination group had greater food-cue reactivity towards unhealthy savory food than the low discrimination group in the caudate, putamen, insula, frontal pole and lateral orbitofrontal cortex (Fig. 2b).

When healthy foods were compared with nonfoods, the high discrimination group had greater food-cue reactivity towards healthy food cues than the low discrimination group in the superior frontal gyrus and middle frontal gyrus (Fig. 2c).

The low discrimination group did not differ significantly from the high discrimination group on the aforementioned contrasts (Table 2).

When unhealthy sweet foods were compared with healthy sweet foods, the high discrimination group had lower food-cue reactivity towards unhealthy sweet foods than the low discrimination group in the ventromedial prefrontal cortex (vmPFC). No significant discrimination-related differences were found in the comparison of unhealthy savory foods versus healthy savory foods.

### Discrimination-related food-cue brain analysis

The Everyday Discrimination Scale (EDS) score correlated positively with greater reactivity to unhealthy sweet food (β = 0.29, *q* = 0.03), unhealthy savory food (β = 0.32, *q* = 0.03) and healthy food (β = 0.72, *q* < 0.001) in the discrimination-related composite food-cue region of interest (ROI).

### Discrimination-related gut-metabolite analysis

**Fig. 3 |:**
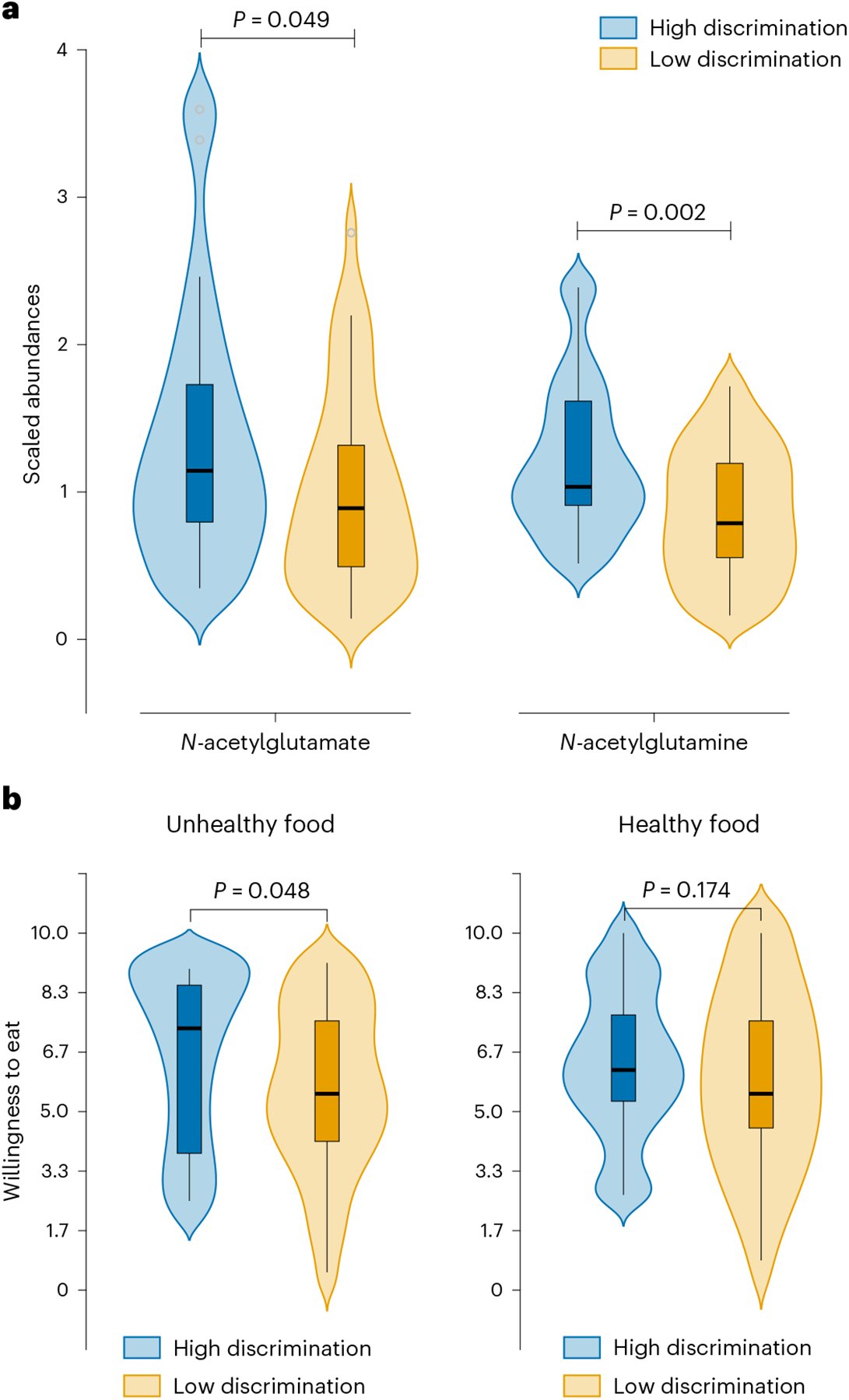
Comparison of the levels of metabolites and willingness to eat for individuals in the high and low discrimination groups. **a**, *N*-acetylglutamate and *N*-acetylglutamine levels in high and low discrimination groups. Glutamate-pathway metabolites were compared between high and low discrimination groups using generalized linear modeling, controlling for BMI, sex, age, race, diet and SES. High discrimination, *n* = 30; low discrimination, *n* = 32. **b**, Willingness to eat ratings for unhealthy and healthy foods. Comparisons between the high and low discrimination groups were made using generalized linear modeling, controlling for BMI, sex, age, race, diet and SES. High discrimination, *n* = 50; low discrimination, *n* = 57. **a**,**b**, The violin plots represent the data distribution. Boxplots: the boxes indicate the 75th (upper horizontal line), median (middle horizontal line) and 25th (lower horizontal line) percentiles of the distribution; the whiskers indicate the range of data falling within a distance of 1.5× the interquartile range. *P* < 0.05 was considered significant (the *Q* value is the adjusted *P* value after FDR correction for multiple comparisons).

Two metabolites from the glutamate pathway, *N*-acetylglutamate (*P* = 0.04) and *N*-acetylglutamine (*P* = 0.002), were present at significantly higher levels in the high discrimination group than in the low discrimination group (Table 3 and Fig. 3a). *N*-acetylglutamine levels remained significantly different between the two groups after multiple correction (*q* = 0.025). Neither *N*-acetylglutamate nor *N*-acetylglutamine were significantly correlated with SES.

### Willingness to eat unhealthy and healthy foods

The high discrimination group had significantly higher ratings of willingness to eat unhealthy foods (*P* = 0.048) relative to the low discrimination group but this difference did not exist for healthy foods (*P* = 0.174; Fig. 3b).

### Structural equation modeling linking discrimination, brain and gut metabolites

**Fig. 4 |:**
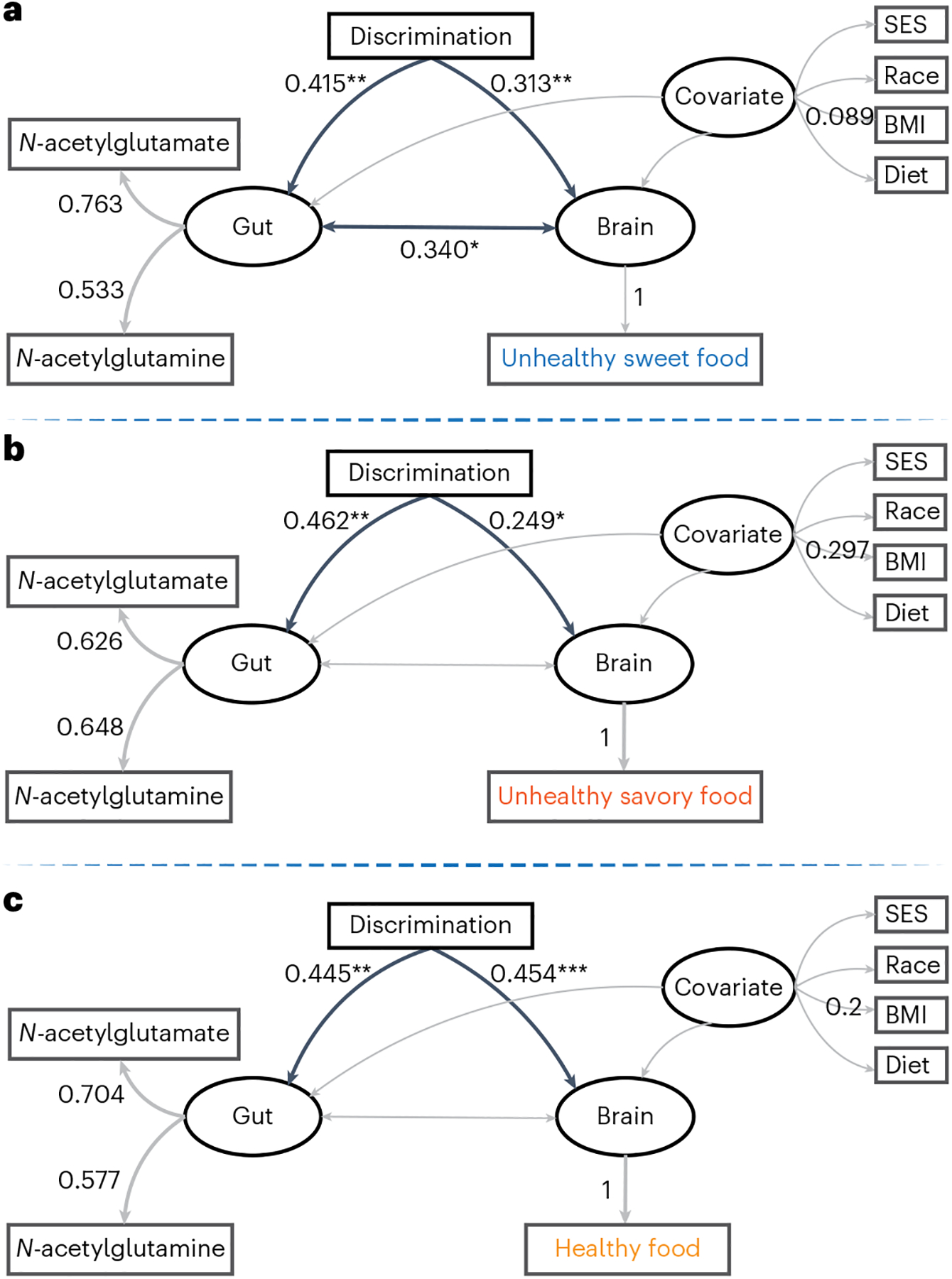
Relationships between discrimination, the discrimination-related brain food-cue responses to different foods and gut metabolites illustrated as SEM models. **a**–**c**, SEM diagrams showing the relationships between brain, gut and discrimination (controlling for BMI, race, diet, and SES) for unhealthy sweet foods (**a**), unhealthy savory foods (**b**) and healthy foods (**c**). Single-headed arrows represent regression and double-headed arrows represent correlation. The values on arrows between latent and observed variables and between latent and observed indicators represent standardized path coefficients and factor loadings (that is, standardized coefficients), respectively. For the arrows between brain, gut and discrimination, black arrows indicate significant correlation or regression coefficients and gray arrows indicate non-significant correlation or regression coefficients. Model fit was assessed using the following indices and criteria: CFI > 0.9, RMSEA < 0.08, GFI > 0.9 and SRMR < 0.08. High discrimination, *n* = 50; low discrimination, *n* = 57. *P < 0.05; **P < 0.01; ***P < 0.001.

In the unhealthy sweet food model (Fig. 4a), positive associations were observed between high discrimination exposure and brain reactivity (standardized coefficient = 0.31, *P* = 0.009) as well as between discrimination and glutamate metabolism (that is, *N*-acetylglutamate and *N*-acetylglutamine levels; standardized coefficient = 0.42, *P* = 0.004). The bidirectional association between the brain and gut was significant (standardized coefficient = 0.34, *P* = 0.048). In terms of the structural equation model (SEM) fit, the root mean square error of approximation (RMSEA) was 0.0, comparative fit index (CFI) was 1.0, goodness-of-fit index (GFI) was 0.955 and standardized root mean square residual (SRMR) was 0.071; all indices suggested a good fit.

For the unhealthy savory food model (Fig. 4b), discrimination correlated positively with brain reactivity (standardized coefficient = 0.249, *P* = 0.043) as well as between discrimination and glutamate metabolism (standardized coefficient = 0.462, *P* = 0.006). No significant correlations between the brain and gut were observed (standardized coefficient = −0.227, *P* = 0.216). The RMSEA was 0.0, CFI was 1.0, GFI was 0.964 and SRMR was 0.058, suggesting a good model fit.

In the healthy food model (Fig. 4c), positive associations were observed between high discrimination exposure and brain reactivity (standardized coefficient = 0.454, *P* < 0.001) as well as between discrimination and glutamate metabolism (standardized coefficient = 0.445, *P* = 0.004). However, the bidirectional association between the brain and gut was not significant (standardized coefficient = 0.155, *P* = 0.373). The RMSEA was 0.0, CFI was 1.0, GFI was 0.969 and SRMR was 0.058; thus, all indices suggested a good model fit.

## Discussion

We investigated associations between self-reported discrimination exposure and alterations in the BGM system. Discrimination exposure was associated with increased food-cue reactivity in frontal-striatal regions involved in reward processing, motivation and executive control, especially towards unhealthy foods. The alterations in the brain were consistent with observed unhealthy food preference—as indicated by an increased willingness to eat—in individuals who reported higher levels of exposure to discrimination. Discrimination exposure was also associated with altered gut metabolites from the glutamate pathway involved in oxidative stress and inflammation. Complex relationships between discrimination exposure and bidirectional brain–gut alterations were observed, especially when evaluating brain reactivity to unhealthy sweet foods (but not unhealthy savory or healthy foods).

### Discrimination-associated brain food-cue reactivity

Unhealthy food cues elicited greater activation in regions associated with reward processing and appetitive responses (insular cortex, orbitofrontal cortex, inferior frontal gyrus, striatum (caudate and putamen) and frontal operculum) in individuals reporting more discrimination experiences than in those with fewer experiences. These frontal-striatal regions have been linked to food-cue reactivity and play a key role in controlling feeding behavior in response to reward and hedonic aspects of food^54–56^. In contrast, healthy food cues were associated with brain reactivity in the frontal pole, middle frontal gyrus and superior frontal gyrus, which partially overlap with the dorsolateral prefrontal cortex (dlPFC). These regions have been implicated in cravings and executive control. These results suggest that stress may lead to exaggerated brain responses associated with reward processing and motivation as well as compromised frontal processes associated with self-regulation in response to unhealthy foods^18,57–59^.

Individuals with greater exposure to discrimination also had altered brain responses in the superior frontal gyrus, which partially belongs to the dlPFC, a brain region associated with executive control, reward appraisal and food-cue-induced craving modulation^60–62^. A recent study revealed a link between racial discrimination and maladaptive eating behaviors (for example, overeating and loss-of-control eating) in young Black women, potentially as a way to cope with stress^63^. Studies have also shown that those with higher levels of food addiction (that is, addiction-like loss-of-control eating behaviors) exhibited altered food-cue reactivity to unhealthy foods in the superior frontal gyrus^64^. The dlPFC has been implicated in food-choice-related self-control over appetitive food cravings^65,66^ and is associated with more effortful exertion of self-control in response to food-cue-induced cravings in obesity^67^. The enhanced frontal alterations in the high discrimination group might indicate an ineffective (for example, more effortful) regulation of cue-induced cravings, suggesting compromised executive control, particularly for unhealthy foods and even healthy foods with moderate reward value.

The analyses using the composite mask also demonstrated heightened brain food-cue responses in frontal-striatal networks with greater discrimination exposure, suggesting heightened reward processing and compromised executive control in response to unhealthy foods.

### Discrimination-related gut-metabolite alterations

Greater discrimination exposure was associated with higher levels of *N*-acetylglutamate and *N*-acetylglutamine, which are linked to glutamate metabolism. This pathway has been implicated in inflammatory processes and oxidative stress, as well as obesity patho-physiology. Similarly, discrimination is associated with increased systemic inflammation and decreased levels of gut metabolites with anti-inflammatory and cardioprotective properties but only when the sample is stratified according to race/ethnicity^33^. The untargeted analyses conducted in this previous study did not reveal significant changes in the metabolome related to discrimination^33^, which could be attributed to not accounting for key confounding variables. The specific role of *N*-acetylglutamate and *N*-acetylglutamine is still an active area of research but their direct connection to glutamate metabolism suggests potential implications for glutamate levels. *N*-acetylglutamate is both a host- and microbial-derived metabolite, and is altered in patients with chronic obstructive pulmonary disease and progressive inflammatory lung disease^68^. Glutamate metabolism is also implicated in obesity-associated mechanisms. The abundance of gut glutamate-fermenting microbiota (*Bacteroides thetaiotaomicron*) is decreased in individuals with obesity and inversely correlated with serum glutamate levels; weight loss through bariatric surgery can partially reverse such alterations^69^.

The existing literature demonstrates the key role of microbiota-derived metabolites and their derivatives in gut–brain communication^70^. Gastrointestinal metabolite signatures have demonstrated that alterations in the gut microbiota are closely correlated with alterations in gut and brain glutamate levels^71,72^, suggesting gut-modulated CNS glutamatergic neurotransmission. *N*-acetylglutamate may play a role in regulating *N*-acetyl-l-aspartyl-l-glutamate and brain function^73^. *N*-acetyl-l-aspartyl-l-glutamate, a dipeptide that is most abundant in the brain, acts as a neuromodulator at glutamatergic synapses, inhibiting excessive glutamate signaling^74^. *N*-acetylglutamate has also been implicated in the brain sleep–wake cycle^75^. Although the exact role of *N*-acetylglutamate and *N*-acetylglutamine is still under investigation, their direct relationship to glutamate metabolism suggests a potential impact on brain function.

Glutamate, the major excitatory neurotransmitter in the CNS, is a non-essential amino acid associated with numerous stress responses^76^. Inflammation influences the release, transmission and metabolism of glutamate, leading to accumulated extracellular glutamate in the CNS^77^. Prolonged presence of glutamate can induce excitotoxicity and oxidative stress, two of the major mechanisms responsible for neuronal damage^78^. Glutamate may also be involved in the biological mechanisms underlying depression, anxiety-related disorders and obesity risk^42,79,80^. Early life stress is associated with dysregulation of gut glutamate metabolites, potentially through glutamatergic excitotoxicity and increased oxidative stress^44^. Circulating glutamate is also associated with excess abdominal adipose tissue in obesity, which is potentially related to expression of the *GLUL* gene (encoding glutamate-ammonia ligase) and inflammatory genes in adipose tissue^81,82^.

We see evidence for a strong association between gut levels of glutamate metabolites with greater discrimination exposure—this relationship may certainly contribute to potentially excitotoxic sequelae of glutamate and its derivatives as well as a proinflammatory state in obesity.

### Discrimination and communication in the BGM system

The results presented here suggest that unhealthy sweet foods may play a major role in the bidirectional communication between the brain and gut with higher discrimination exposure. The mechanisms underlying this association may involve inflammatory processes in the BGM system involved in stress-induced unhealthy eating behaviors and dysfunctional glutamatergic signaling. Stress could promote unhealthy food choices^83^, particularly for sweet foods^84,85^, which could adversely influence the systemic homeostasis within the BGM system, resulting in inflammation in the CNS and an increased risk of obesity and stress-related neuropsychiatric complications^86–89^. Sugar, delivered intestinally, can activate gut-to-brain pathways by activating vagal neurons in mice, underlying the highly appetitive effects of sugar^53^. Interestingly, racial and ethnic differences in sweet preference have also been observed^90^. Greater desire for sweet taste is associated with higher levels of stress to a greater extent among young Black adults than among similarly aged White adults^91,92^. Accordingly, discrimination, as a stressor, could promote more consumption of sweet food relative to that for highly savory food^93,94^.

Sweet-tasting foods present an analgesic effect; individuals consume more sweet foods after acute physical pain^50^. Furthermore, the consumption of sweet-tasting food increases pain tolerance via the endogenous opioid system in the brain^51^. Extensive research has highlighted variations in the opioidergic system in the context of racial discrimination stress and its effect on pain perception^95,96^. Stress can upregulate the amygdala κ-opioid receptor, inducing dysphoria, and modulate the μ-opioid receptor to regulate reward processes^97,98^. When stress activates the opioidergic system, it could affect the rewarding properties of food and potentially lead to stress-related changes in food choices, eating behaviors and obesity. Hence, future studies should further examine the role of the opioidergic system in the context of the effects of discrimination on brain–gut communication, specifically focusing on its influence on unhealthy food preferences, particularly sugary foods. In addition, discrimination together with the disproportionate level of exposure to targeted marketing of unhealthy foods (especially high-fat high-sugar foods) in Black and Latino consumers may exacerbate the adverse health effects and worsen health disparities^99,100^.

We found that individuals who experienced more discrimination showed a decreased reactivity in the vmPFC when exposed to unhealthy sweet food but this was not observed with the unhealthy savory foods. It is probable that the observed difference is specific to the sweet feature. The vmPFC, along with the adjacent medial part of the orbitofrontal cortex, encodes the pleasantness or value of taste and flavor^101,102^, including the perception of carbohydrate content in food cues^103^. Exposure to stress is associated with attenuated sweet taste^104^. African Americans have shown heightened and sustained desire for intense sweet tastes, as well as greater perceived stress, relative to White Americans^91^. It is also possible that this attenuation of sweet perception is a result of increased consumption of sweet food, as higher dietary sugar intake has been found to decrease the perceived sweetness of sweet food^105^.

The observed discrimination-related BGM disruptions may be associated with an unhealthy diet. Perceived day-to-day racial discrimination is known to be linked to unhealthy eating habits^106^, which can trigger inflammation in the BGM system, as implicated in obesity pathogenesis^86^. A high-fat high-sugar diet could alter gut microbiome diversity and increase Gram-negative bacteria rich in endotoxin lipopolysaccharides as well as increase gut permeability, increasing the translocation of lipopolysaccharides across the intestinal epithelium and promoting local and CNS inflammation^86^. In addition to inflammation, oxidative stress and dyslipidemic processes, high-fat-diet-induced obesity is linked to altered brain neurotransmitter glutamate levels in rats^43^. Stress and stress-induced unhealthy eating have a detrimental effect on the glutamatergic system. Inflammatory cytokines can influence glutamate metabolism through effects on astrocytes and microglia^80^. Modulation of glutamatergic receptor activity along the BGM axis may influence gut and brain functions and participate in the pathogenesis of local and brain disorders, such as anxiety and depressive disorders^107^.

In this study glutamate metabolites were associated with greater neural response to food cues in the frontal-striatal network with higher discrimination exposure. The frontal-striatum (limbic) network is driven by glutamatergic and dopaminergic neurotransmission in humans^48^. Frontal glutamate plays an important role in reward-guided decision-making in humans^108^ and modulates fronto-limbic connectivity^109^. In mice, a high-fat high-sugar diet alters glutamate transmission in the dorsal striatum, a core region implicated in food motivation and reward processing^110^. Given the limited literature on the exact role of specific glutamate metabolites, further research is warranted to elucidate the finer and more direct associations between these metabolites and their role in communicating with the brain.

### Limitations and future directions

Some of the limitations of this study, in which discrimination-related differences in neural responses to food cues and gut metabolites were examined, merit consideration. Although we controlled for sex in the analyses, we acknowledge that men were under-represented. Previous studies suggested that gut-induced alterations in CNS neurochemicals may be sex-specific^111,112^. Our study did not have sufficient samples of specific racial/ethnic groups to conduct stratified analyses by group. Therefore, future research with larger and more balanced samples should attempt to replicate these results and explore the potential moderating effects of sex and race/ethnicity and source of discrimination. Finally, this was a correlational study; accordingly, longitudinal studies are needed to explore the causal effects of discrimination exposure and altered BGM signatures.

## Conclusions

In this study we have elucidated the impact of self-reported discrimination on brain food-cue reactivity and gut microbiome interactions utilizing a systems-biology approach. We demonstrated that experiences of discrimination lead to disruptions in the BGM system, with altered neural response to food cues in regions associated with reward processing and executive control as well as gut glutamate metabolites implicated in stress and inflammation. These alterations may confer vulnerability to obesity and obesity-related comorbidities in individuals with more exposure to discrimination. Thus, brain-targeted treatments (for example, brain stimulation) that could dampen an overactive food-reward system or enhance frontal control could potentially be used as a neuromodulatory tool to normalize altered brain circuits associated with discrimination exposure^113^. It is also possible to target glutamatergic pathways, such as by a probiotic supplement or Mediterranean diet with anti-inflammatory benefits, as a therapeutic approach for the treatment of stress-related experiences such as discrimination^114–116^.

## Methods

### Study participants

The study group was comprised of 107 individuals (87 women) recruited from the Los Angeles community through advertisements and local clinics. Peri- and post-menopausal women were excluded, as determined by the self-reported last day of the previous cycle, and enrolled women were scanned during the follicular phase of the menstrual cycle. Participants were excluded if they had any major medical/neurological conditions, current or past psychiatric illnesses, comorbidities such as vascular disease or diabetes, weight loss/abdominal surgeries, substance-use disorders, tobacco dependence (half a pack or more daily) or metal implants and if they used medications that interfere with the CNS, regularly used analgesics, were pregnant or breastfeeding, or performed extreme strenuous exercise (>8 h of continuous exercise per week). Participants whose weight exceeded 181 kg (400 pounds) were excluded due to weight constraints of the MRI scanner.

All procedures complied with institutional guidelines and were approved by the Institutional Review Board at UCLA’s Office of Protection for Research Subjects. All participants provided written informed consent.

Participant data included BMI, race, age, sex, SES^117^ and diet (Supplementary Methods and Supplementary Table 1). Diet was categorized into standard or nonstandard American diet based on self-reported questionnaires where participants report which diet is consumed on a regular basis in our analyses as defined in previous studies (Supplementary Table 1)^33^. Multimodal data, including functional MRI imaging (fMRI), fecal metabolomics and clinical and behavioral measures were also collected.

### Clinical and behavioral assessments

Participants completed the validated and widely used EDS^118^, which measures chronic experiences of unfair treatment^118–120^. The EDS is a validated and widely used measure that captures chronic experiences of unfair treatment in various domains of life. The EDS does not specifically target discrimination based on race, gender, age or poverty but rather assesses overall experiences of discrimination in daily life. For example, one of the questions in the EDS asks participants ‘In your day-to-day life, how often do any of the following things happen to you? You are treated with less courtesy than other people are.’. This demonstrates that our measure of discrimination is not limited to any specific type but encompasses a broad range of unfair treatment experiences. Because there is no consensus on the cut-offs of the EDS, scores were dichotomized to categorize participants into two groups—high discrimination exposure (EDS > 10, *n* = 50) and low discrimination exposure (EDS ≤ 10, *n* = 57)—based on the median score of this sample, as used in previous studies^33,120–122^. Participants were excluded if their EDS score was zero due to their distinct nature in reporting discrimination (unwilling or unable to report)^123^.

### Baseline characteristics

Baseline demographic and clinical characteristics were compared in the R software^124^ using a Student’s *t*-test for continuous variables and a *χ*^2^ test for categorical variables. A two-way analysis of variance was conducted to examine the interaction effect between discrimination groups (high versus low discrimination) and dietary style (American versus non-American) on BMI.

### Food-cue task-MRI acquisition, processing and analyses

Brain data were acquired using a 3.0 T Prisma MRI scanner (Siemens); acquisition details are provided in Supplementary Methods. Participants were asked to fast for approximately 6 h previous to scanning and this was confirmed by the study coordinator before scanning took place. Participants completed the food-cue task in the scanner to evaluate neural responses to different types of foods. Pictures were organized into five groups: unhealthy (high calorie) savory, unhealthy (high calorie) sweet, healthy (low calorie) savory, healthy (low calorie) sweet and nonfood, comprising pixelated images created from food pictures (as a control comparison). All food images were uploaded to the E-prime software^125^; half were copied and pixelated to control for color, brightness and contrast. Images were arranged into blocks of six, comprising either unaltered or pixelated images only, with a total of 18 blocks. Each image was shown for 3 s. A black screen with a white crosshair was displayed for 12 s before the first block of images, between each block of images and after the final block of images. Two slideshows (order 1 and order 2) were created using the same 18 blocks of images arranged in different orders. Participants watched both sets of images in the scanner.

At the end of the scan, participants reported their willingness to eat the food items they saw in the scanner by answering the question ‘How much do you want to eat what you just saw?’. The response options ranged from zero (not at all) to ten (very much).

Neuroimaging data were processed using the fMRI Expert Analysis Tool (FEAT; version 6.0) included in the FMRIB Software Library (FSL)^126^. Preprocessing included motion correction, brain extraction, 100-s high-pass filtering and spatial smoothing with a 5-mm full-width at half-maximum Gaussian kernel. Functional data were aligned to the structural image of each participant and then registered into Montreal Neurological Institute (MNI) standard space using affine transformation through FSL’s Linear Image Registration Tool (FLIRT).

### Whole-brain analysis

To determine discrimination-related differences in food-cue reactivity towards specific food types, we specified the following contrasts: (1) unhealthy sweet food versus nonfood, (2) unhealthy savory food versus nonfood, (3) healthy food versus nonfood, (4) unhealthy sweet food versus healthy sweet food and (5) unhealthy savory food versus healthy savory food. The corresponding reversed contrasts were specified. For each participant, ten contrast maps were created in the first-level analysis, which were then imputed into random-effects group-level analyses using FSL’s Local Analysis of Mixed Effects (FLAME1) in a whole-brain analysis with outlier de-weighting. Group-level (high versus low discrimination) unpaired Student’s *t*-tests were performed in FEAT using a mixed-effects model with BMI, age, sex, race, diet and SES as covariates. All statistical maps were family-wise error cluster-corrected for multiple comparisons (cluster height threshold, *Z* > 2.3; cluster significance, *P* < 0.05).

### Discrimination-related food-cue ROI analysis

Significant clusters in the contrasts from the whole-brain analysis (high versus low discrimination) were combined to create a discrimination-related food-cue ROI mask. Brain signal changes (β values from the first-level statistical models) were extracted separately for each participant. Multiple linear regression analyses were conducted to test the effect of discrimination exposure on brain signal change in the composite food-cue ROI for each contrast, adjusting for BMI, age, sex, race, diet and SES, and correcting for multiple comparisons using the false discovery rate (FDR) according to the Benjamini–Hochberg procedure^127^. This step was used to confirm the robustness of discrimination-related food-cue activity in a linear fashion, and the brain signal extracted from the composite food-cue ROI was further used in an SEM analysis.

### Fecal metabolites collection, processing and analyses

Fecal collection and processing was conducted on a subsample of participants (*n* = 62) as previously described^128^ and detailed in Supplementary Methods. The fecal samples were stored at −80 °C and shipped to Metabolon for processing and analysis as a single batch on their global metabolomics and bioinformatics platform using ultrahigh-performance liquid chromatography and tandem mass spectrometry^129^. Raw data were curated by mass spectrometry using specialized software as previously described^129^. The amount of missing data was low (<3%). However, missing values of raw data were filled using the median value and ineffective peaks were removed through the interquartile range denoising method. In addition, the internal standard normalization method was employed in the data analysis. A dataset for multiple classification analysis was compiled from the metabolite profiling results and a three-dimensional matrix involving metabolite numbers, sample names and normalized peak intensities was used as input to the MetaboAnalyst web software version 3.0 (http://www.metaboanalyst.ca). Because of our a priori interest in the metabolites from the glutamate pathway associated with the processes of stress, only glutamate metabolites were included in our analyses.

### Gut metabolites

Twelve metabolites from the glutamate pathway were compared between the high and low discrimination groups using generalized linear modeling, controlling for BMI, age, sex, race, diet and SES. Multiple comparisons were corrected for using the FDR method^127^. Pearson’s correlations were used to assess the associations between the gut metabolites and key psychosocial variables (for example, SES) that showed significant differences between the high and low discrimination groups.

### Willingness to eat unhealthy and healthy foods

The willingness of participants to eat based on the ratings of unhealthy or healthy food was compared separately between the high and low discrimination groups using generalized linear modeling, controlling for BMI, sex, age, race, diet and SES.

### SEM

The Lavaan package in R^130^ was used to perform SEM modeling. One latent variable was created for gut metabolites affected by discrimination exposure, as determined in the initial analyses. Three models were developed to illustrate the pathways that link EDS scores and discrimination-related brain and gut signatures. The unhealthy sweet food model included neural reactivity to unhealthy sweet food cues (versus nonfood) extracted from the composite food-cue ROI as the brain feature. The unhealthy savory food model included neural reactivity to unhealthy savory food cues (versus nonfood) extracted from the composite food-cue ROI as the brain feature. The healthy food model included neural reactivity to healthy food cues (versus nonfood) extracted from the composite food-cue ROI as the brain feature. Glutamate metabolites found to differ significantly between the high and low discrimination groups were included as the gut feature. We controlled for BMI, race, diet and SES as covariates. Model fit was assessed using the following indices and criteria: CFI > 0.9, RMSEA < 0.08, GFI > 0.9 and SRMR < 0.08 (ref. 131). The significance level was set at *P* < 0.05 for all SEM statistical significance testing.

## Supplementary Material

supplemental material

## Figures and Tables

**Fig. 1 |:**
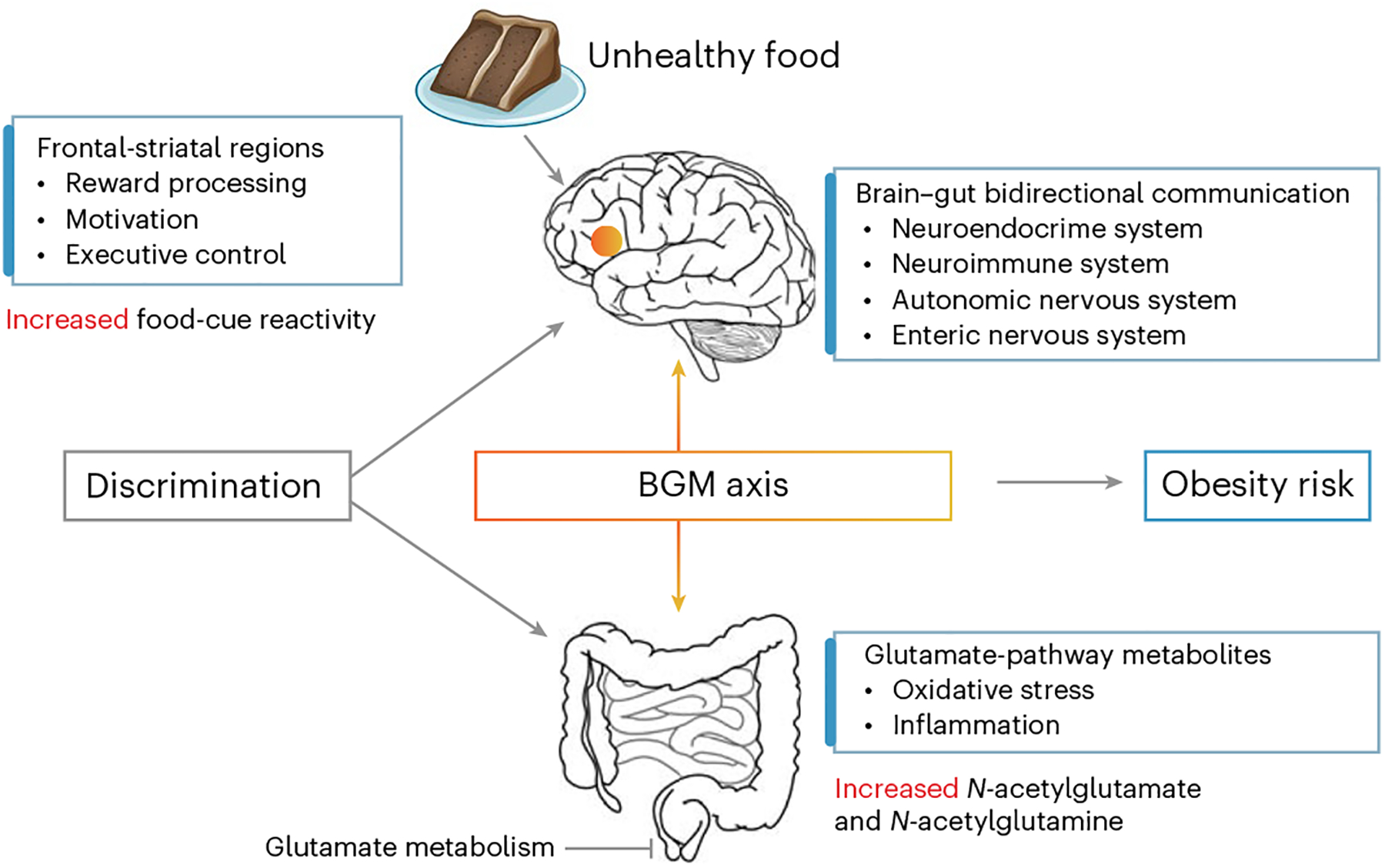
Discrimination exposure increases risk for obesity by disrupting the brain gut system. Discrimination exposure is associated with increased food-cue reactivity—especially towards unhealthy sweet foods—in the frontal-striatal regions involved in reward processing, motivation and executive control, as well as altered gut metabolites in the glutamate pathway associated with oxidative stress and inflammation.

**Table 1 |:** Participant characteristics

Parameter	All participants	High discrimination group (*n* = 50)	Low discrimination group (*n* = 57)	*P* value
Age (yr), mean (s.d.) [range]	28.83 (9.93) [18–54]	28.06 (10.15) [18–54]	29.51 (9.76) [18–54]	0.46
Sex, *n* (%)				0.75
Women	87 (81.31%)	40	47	
Men	20 (18.69%)	10	10	
BMI (kg m^−2^), mean (s.d.) [range]	30.99 (6.10) [18.00–47.54]	30.99 (6.61) [18.10–45.28]	30.99 (5.68) [18.00–47.54]	0.999
Dietary style, *n* (%)				0.3
Standard American diet	55 (51.40%)	26	29	
Nonstandard American diet	50 (46.73%)	22	28	
Missing data	2 (1.87%)	2	0	
Ethnicity, *n* (%)				0.65
Hispanic	57 (53.27%)	26	31	
White	15 (14.02%)	6	9	
Black	11 (10.28%)	4	7	
Asian	15 (14.02%)	8	7	
Other	9 (8.41%)	6	3	
Educational level, *n* (%)				0.58
Less than a high school diploma	1 (0.93%)	0	1	
High school graduate	6 (5.61%)	2	4	
Some college (no degree)	33 (30.84%)	19	14	
College graduate	33 (30.84%)	14	19	
Graduate school	27 (25.24%)	11	16	
Missing data	7 (6.54%)	4	3	
Annual income ($), *n* (%)				0.52
≤19,000	11 (10.28%)	7	4	
20,000–49,000	26 (24.30%)	13	13	
50,000–79,000	26 (24.30%)	9	17	
≥80,000	31 (28.97%)	14	17	
Missing data	13 (12.15%)	7	6	
Marital status, *n* (%)				0.61
Never married	70 (65.42%)	33	37	
Married	23 (21.50%)	9	14	
Divorced	6 (5.61%)	4	2	
Widowed	1 (0.93%)	0	1	
Missing data	7 (6.54%)	4	3	
SES^a^, mean (s.d.) [range]	5.73 (1.32) [3–8]	5.35 (1.44) [3–8]	6.07 (1.13) [4–8]	0.01*

Student’s *t*-tests for continuous variables and *χ*^2^-squared tests for categorical variables were performed; a two-way analysis of variance was conducted to examine the interaction effect between discrimination groups (high versus low discrimination) and dietary style (American versus non-American) on BMI;

* *P* < 0.05.

a SES was measured using the MacArthur Scale of Subjective Social Status.

**Table 2 |:** Significant clusters from whole-brain analysis of food-cue reactivity comparing high and low discrimination groups

Contrast high>low discrimination	Cluster region(s)	*X* (MNI)	*Y* (MNI)	*Z* (MNI)	Maximum *Z*
Unhealthy sweet food versus nonfood	L insula	−26	20	−6	3.70
	L insula	−24	26	10	3.67
	L inferior frontal gyrus, pars opercularis	−42	18	10	3.63
	L lateral orbitofrontal cortex	−26	22	−2	3.51
	L inferior frontal gyrus, pars opercularis	−32	16	24	3.35
	Frontal operculum	−34	14	16	3.35
Unhealthy savory food versus nonfood	R orbitofrontal cortex	20	34	−12	3.94
	R orbitofrontal cortex	22	38	−12	3.93
	R frontal pole	22	38	−18	3.75
	R putamen	22	20	2	3.61
	R caudate	14	22	−4	3.52
	R insular	26	26	2	3.32
Healthy food versus nonfood	R middle frontal gyrus (dlPFC)	24	28	34	4.08
	R superior frontal gyrus (vmPFC)	22	64	24	3.89
	R middle frontal gyrus (dlPFC)	36	8	40	3.86
	R superior frontal gyrus (dlPFC)	20	18	54	3.82
	Frontal pole (dlPFC)	28	44	36	3.64
	R middle frontal gyrus (dlPFC)	24	32	40	3.58
Unhealthy sweet food versus healthy sweet food	vmPFC	24	56	28	3.65
Unhealthy savory food versus healthy savory food	Not significant				

Family-wise error cluster level correction: *Z* > 2.3, *P* < 0.05. Peak voxel coordinates are in MNI space. Results were controlled for BMI, sex, age, race, diet and SES. High discrimination, *n* = 50; low discrimination, *n* = 57. L, left hemisphere; R, right hemisphere.

**Table 3 |:** Comparisons in glutamate metabolites between high and low discrimination groups

Glutamate-pathway metabolite	*T* value	*P* value	FDR-adjusted *P* value
Carboxyethyl-GABA	−0.3283	0.7437	0.8666
GABA	−1.2034	0.2329	0.5575
Glutamate	0.4368	0.6636	0.8666
Glutamate, γ-methyl. ester	0.2616	0.7944	0.8666
Glutamine	0.1364	0.8919	0.8919
*N*-acetyl-l-aspartyl-l-glutamate	−1.8042	0.0755	0.3020
***N*-acetylglutamate**	**2.0038**	**0.0490**	0.2938
***N*-acetylglutamine**	**3.2011**	**0.0021**	**0.0247**
*N*-methyl-GABA	1.1242	0.2648	0.5575
*N*-methylglutamate	−0.7593	0.4502	0.7237
Pyroglutamine	−0.7061	0.4824	0.7237
Succinylglutamine	−1.0916	0.2788	0.5575

Generalized linear modeling was used to compare the levels of 12 metabolites from the glutamate pathway between the high and low discrimination groups. The *Q* value is the adjusted *P* value after FDR correction for multiple comparisons (shown in bold). High discrimination, *n* = 30; low discrimination, *n* = 32. GABA, γ-aminobutyrate.

## Data Availability

De-identified individual participant data (brain) can be shared on request and will be made available through the Center’s pain repository portal (https://www.painrepository.org/). To access the data, participants will fill out a user agreement, following which access to the data will be made available through a secure password-protected portal. The raw microbiome sequences can be accessed at NIH NCBI BioProject (BioProject ID: PRJNA946906).
