# Supplementary material for: Discrimination exposure impacts unhealthy processing of food cues: crosstalk between the brain and gut
Source: Nat Ment Health. Author manuscript; Available in PMC 2023 Dec 13. (PMC10718506; doi:10.1038/s44220-023-00134-9)
Supplement: supplemental material [file NIHMS1947980-supplement-supplemental_material.docx]

**Supplementary Information for**

**Discrimination Impacts Unhealthy Processing of Food-Cues: The Crosstalk between the Brain and Gut**

**Supplementary Methods**

*Socioeconomic status* (SES) was measured using The MacArthur Scale of Subjective Social Status which is a widely used measure of subjective social status (48).

*Diet Questionnaires:* All participants completed the UCLA Diet Checklist, which is a questionnaire developed by our institution, intended to represent the diet that best reflects what the participant consumes on a regular basis. The specific diets incorporated into this checklist are summarized in supplemental **Table S1**. Participants were also allowed to choose “other” if they felt they consumed a diet that was distinct from the ones listed on the Diet Checklist. Our institution’s Diet Checklist has been internally validated against the standardized Diet History Questionnaire (DHQ) III. For data analysis, we combined the standard and modified American diets as one category. Mediterranean, vegan, vegetarian, and gluten-free were categorized individually, and all other diets were combined as “other” for analysis.

***Brain Magnetic Resonance Imaging***

Whole-brain structural and functional data was acquired using a 3.0T Siemens Prisma MRI scanner (Siemens, Erlangen, Germany). Additional detailed information on the standardized acquisition protocols and quality control measures are provided in previously published studies (Dong, Gupta, et al., 2020; Dong, Mayer, et al., 2020; Gupta, Mayer, Acosta, et al., 2017; Gupta, Mayer, Hamadani, et al., 2017; Gupta et al., 2018; Labus et al., 2015; Osadchiy et al., 2019; Osadchiy et al., 2020).

*Structural MRI Acquisition*: High-resolution T1-weighted images were acquired: echo time/ repetition time (TE/TR)=3.26ms/2200ms, field of view=220×220mm, slice thickness=1mm, 176 slices, 256×256 voxel matrix, and voxel size=0.86×0.86×1mm.

*Functional MRI Acquisition*: Whole-brain scans were acquired with Participants breathing and blinking normally while watching the slideshow, using an echo planar sequence with the following parameters: TE/TR=28ms/2000ms, flip angle=77º, scan duration=10m6s, FOV=220mm, slices=40, and slice thickness=4.0mm.

**Fecal Metabolomics Collection and Processing**

Raw data was curated by mass spectrometry using specialized software as previously described (Evans et al., 2009). The number of missing data was low (<3%). However, missing values of raw data were filled using the median value, and ineffective peaks were removed through the interquartile range denoising method. In addition, the internal standard normalization method was employed in the data analysis. The dataset for the multiple classification analysis was compiled from the metabolite profiling results and a 3D matrix involving metabolite numbers, sample names, and normalized peak intensities were fed into the MetaboAnlyst web software 3.0 ([http://www.metaboanalyst.ca](http://www.metaboanalyst.ca/)).

**Table S1**

*Diet checklist categories*

| **Diet Category** | **Components** |
| --- | --- |
| Standard American | High consumption of processed foods, pastas, and breads. Meats, including red meat, fish, eggs, and dairy products consumed.  Vegetables and fruits consumed, but not in large quantities |
| Modified American | High consumption of processed foods, pastas, and breads (mainly whole grain). Poultry, fish, eggs, and dairy products consumed. Red meat consumed in limited quantities  Vegetables and fruits consumed, but not in large quantities |
| Mediterranean | High consumption of fruits, vegetables, bread and other cereals, beans, nuts, and seeds.  Olive oil is the key monounsaturated fat source.  Dairy products, fish, and poultry are consumed in low to moderate amounts. Little red meat is consumed. Eggs are eaten zero to four times a week and wine is drunk in moderate (or low) amounts. |
| Paleo | Consumption of basic foods such as plain meat, fish, shellfish, eggs, nuts, vegetables, fruits, berries, and mushrooms.  Minimally processed oils, such as avocado, olive or coconut oil, are used for cooking.  Dairy products, legumes, dry beans, grains, coffee, alcohol, sugar, and processed foods are excluded. |
| Vegan | Focus is on plant-based foods. Includes fruits, vegetables, dried beans and peas, grains, seeds, and nuts. Excludes all meat and animal products. |
| Vegetarian  (6 categories) | Focus is on plant-based foods. Includes fruits, vegetables, dried beans and peas, grains, seeds, and nuts. |
| Vegetarian | Diet excludes all meat but will allow animal-derived ingredients, i.e. honey and gelatin |
| Lacto-Vegetarian | Diet includes plant foods plus dairy products, no eggs |
| Ovo-Vegetarian | Diet includes plant foods plus eggs, no dairy |
| Lacto-Ovo-Vegetarian | Diet includes both dairy products and eggs |
| Pescatarian | Diet includes fruits, vegetables, dried beans and peas, grains, seeds, and nuts. Excludes all meat except fish. |
| Raw Vegan/ Raw Food | Consumption of unprocessed vegan foods that have not been heated above 115 degrees Fahrenheit (46 degrees Celsius) |
| Gluten-Free | Diet includes most foods but avoids the protein, gluten, which is found in wheat, barley, and rye. |
| Dairy-Free | Diet includes most foods but avoids dairy. |
| Low FODMAP (Fermentable Oligo-,Di-, Monosaccharides, and Polyols) | Diet limits foods high in sugar and carbohydrates (fructose, lactose, fructans, galactans, and polyols). |
